# Prediction of high-dose regions in the jaw as a basis for decision-making in dental rehabilitation prior to radiotherapy in the head and neck area

**DOI:** 10.1016/j.ctro.2026.101152

**Published:** 2026-03-21

**Authors:** Ingmar Staufenbiel, Knut Adam, Marco Flohr, Joern Wichmann, Hans Christiansen, Nadine Schlueter, Liv Julia Clare Moog, Robert Blach, Kirstin Vach, Roland Merten

**Affiliations:** a Hannover Medical School, Department of Conservative Dentistry, Periodontology and Preventive Dentistry, Hannover, Germany; b Hannover Medical School, Department of Radiotherapy, Hannover, Germany; c Radiological Practice Marstall, Department of Radiotherapy, Hannover, Germany

**Keywords:** Osteoradionecrosis, Radiation exposure, Dose-adapted dental rehabilitation, Decision tree

## Abstract

**Background and purpose:**

Radiotherapy to the head and neck can lead to severe side effects including xerostomia, caries, and in worst cases to osteoradionecrosis. Dental rehabilitation prior to radiotherapy is strongly recommended to reduce osteoradionecrosis risk, ideally in a dose-dependent manner. However, dentists do not know where high radiation doses are expected and radical tooth extractions continue to be performed. Study aim was to determine the radiation exposure in different dental regions depending on the tumour location to create a decision tree for clinicians.

**Methods:**

The present retrospective study included data of 112 irradiated patients, from which the mean radiation exposure in various dental regions (anterior, intermediary, posterior) of both jaws (upper, lower) was calculated depending on the radiotherapy concept (definitive, adjuvant) and the region of the tumour to be irradiated (oral cavity, oropharynx, hypopharynx, larynx) resulting in 48 subgroups of dental regions. Statistics: linear mixed models with patient as random factor for group differences; multiple testing: Scheffé correction, α = 5%.

**Results:**

Regarding the location, in particular irradiation of oral cavity and oropharyngeal tumours appears to lead to higher radiation exposures, same applies for definitive radiotherapy concepts and the molar region. Considering a radiation exposure of < 40 Gy as the threshold for low risk and > 50 Gy for high risk for severe side effects, of the 48 dental regions 8/10/30 were at high/medium/low risk.

**Conclusion:**

In cases where medium/low risk is expected, radical extraction regimes should be reconsidered. Based on the results, a decision tree for dental rehabilitation planning was developed.

## Highlights

• current recommendations do not consider radiation dose in different dental regions.
• in many cases radical tooth extraction is still performed prior to radiotherapy.
• the mean radiation exposure varied considerably between different dental regions.
• study data were used to create a decision tree with clinical recommendations.
• often no radical tooth extraction is required according to the radiation dose.

## Introduction

The Global Burden of Disease Study 2020 revealed 23.6 million new cancer cases and 10 million cancer deaths globally [1], [2]. This represents a 26.3% increase of new cases compared to the data from 2010. Oesophageal, laryngeal, lip, and oral cavity cancers represent a relevant part with an age-standardized incidence rate of 13.5 per 100′000 patients.

Besides surgical resection, a combined (chemo-)radiotherapy in definitive and adjuvant radiotherapy are usually used as a therapeutic approach. Radiotherapy with ionizing radiation in the head and neck area is often accompanied by adverse side effects. This can cause short and long-term damage and considerable reduction in quality of life [3]. Typical short-term side effects during radiotherapy include mucositis, which can impair oral hygiene and food intake leading up to reduced calorie intake and dehydration. Long-term effects due to damage of salivary gland tissue are hyposalivation accompanied by changes in salivary pH and buffering capacity, microbial colonisation, and higher risk for fast developing radiation caries [4], [5]. However, the most severe side effect of radiotherapy in the head and neck area is osteoradionecrosis, characterized by a mucosal defect with exposed bone not healing within three months [6]. The incidence of osteoradionecrosis decreased significantly due to intensity-modulated radiotherapy (IMRT) and is estimated at less than 5% [7], [8]. It is therefore a rare but debilitating long-term side effect that severely impairs quality of life [9], [10], general health and, in the worst cases, the integrity of the affected jaw. Therapy of osteoradionecrosis ranges from conservative management, including antibiotics, analgesics, oral hygiene to surgical resection with reconstruction. Since osteoradionecrosis usually occurs post-irradiation due to periodontal or periapical inflammation or tooth extraction [11], dental rehabilitation is strongly recommended before starting the radiotherapy, which includes the treatment of cavitated carious lesions, apical inflammation or periodontal disease, often leading to multiple tooth extractions to rule out subsequent risks of osteoradionecrosis [12].

If possible, planning of rehabilitation and reduction of risk for osteoradionecrosis should be done in a dose-dependent manner [13]. The risk of osteoradionecrosis increases immensely at radiation exposure in the bone above 50 Gy [14]. Accordingly, radical dental rehabilitation should be performed in regions where such a high radiation dose is expected including extraction of teeth with high risk of severe inflammation. In the range between 40 and 50 Gy the risk for such sequelae is moderate; below a threshold of 40 Gy, the risk is considered to be low; mostly no teeth need to be extracted and treatments can be planned comparable to healthy persons [4].

Therefore, for sufficient planning of the dental rehabilitation [15], [16], a clear demarcation between regions with different radiation doses would be helpful. The mandatory CT-based 3D-planning of the target volume allows for dosimetry within the different regions of the jaw, which could potentially be used for risk adapted rehabilitation planning. However, dental treatment should not delay the start of radiation. Therefore, waiting for 3D-planning is not possible in most cases. As a consequence, it would be advantageous to develop an atlas providing radiation doses in different jaw regions depending on the tumour location and radiotherapy concept. This study aims to detect whether there are differences in radiation exposure in different jaw and dental regions between individual tumour locations in head and neck cancer and whether it is possible to generate a decision model (tree) facilitating the planning for dental rehabilitation before radiotherapy.

## Patients and Methods

The present retrospective study was conducted in accordance with the “REporting of studies Conducted using Observational Routinely-collected health Data” (RECORD) statement and checklist [17]. The study was approved by the Institutional Review Board (Ethical Committee of Hannover Medical School, ethics vote no. 9693_BO_K_2021).

### Participants and setting

Initially, patients were identified who underwent radiotherapy for head and neck cancer at the Department for Radiotherapy and Special Oncology at Hannover Medical School between 2016 and 2020. All patients admitted to any clinic of Hannover Medical School are asked whether their data may be used for study purposes (standardized consent form for research transfer). Only data from patients who had signed the form were analysed in pseudonymized form. Inclusion criteria: 1) squamous cell carcinoma, 2) bilateral radiation, 3) radiotherapy with a curative approach. Exclusion criteria: edentulism in one or both jaws and large resection-associated bone defects.

**Fig. 1:**
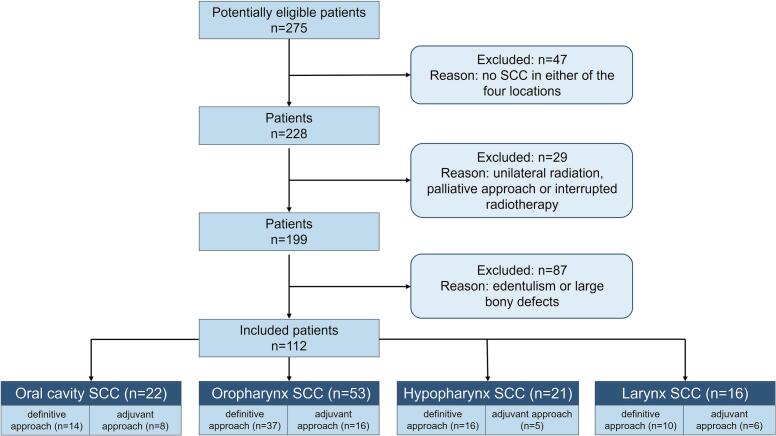
Flow-chart illustrating eligibility criteria, reasons for exclusion, and group assignment according to tumour location and radiotherapy concept of patients included. (SCC: squamous cell carcinoma).

Groups were formed according to location of the tumour (oral cavity, oropharynx, hypopharynx, larynx) and radiotherapy concept (definitive, adjuvant). Information about the excluded patients and group assignment is given in Fig. 1.

All patients received intensity-modulated radiotherapy (IMRT). Depending on the radiotherapy concept, the total/single dose were 66.0 Gy/2.20 Gy for the definitive approach and 62.4 Gy/2.08 Gy for the adjuvant approach. This moderate hypo–fractionation corresponds to the established clinical standard at the investigators' institution, with an eqd2-adapted total dose of 70 Gy for definitive treatment and 63 Gy for adjuvant treatment. The lymphatic drainage pathways were irradiated with a single dose of 1.8 Gy, the primary tumor with a simultaneous integrated boost. Almost all teeth were not within the Planning Target Volume (PTV) of the boost for the primary tumor, but in peripheral isodoses. Therefore, a radiobiological conversion according to eqd2 could be omitted. The absorbed dose was evaluated. The dental regions marked as Volumes of Interest (VOI) were only 2.4 cm3 in size on average. The Dmean, D0.1 cm3, and D0.01 cm3 were determined. Because there were only minor differences, the statistical differences were calculated based on the Dmean. For planning the radiotherapy, each patient underwent a helical CT-scan with a secondary reconstruction interval of 3 mm.

### Measurements

In the planning CT of each patient different tooth compartments were contoured. Three dental regions were defined in both the upper and the lower jaw: the posterior region (molars on the right and left side, wisdom teeth excluded), the intermediary region (canine and premolars on the right and left side), and the anterior region (all incisors).

As this study’s focus was on osteoradionecrosis, the dental alveolus was contoured on the planning CT. For details of contouring process see Supplementary Table 1. The average doses (D_mean_) for the contoured volumes were calculated using Monaco treatment planning system (Software Version 5.11.03 TPS, Elekta, Stockholm, Sweden).

### Statistical methods

The sample size calculation was based on the consideration that the dangers of the radiation dose are usually divided into groups with a difference of 10 Gy. In order to estimate the D_mean_ with a 95% range of 10, 16 patients per group were required with an assumed SD of 10 Gy.

For descriptive analysis median, mean values, and standard deviations were computed for each subgroup with regard to tumour location, radiotherapy concept, and dental region. To test D_mean_ for group differences linear mixed models with patient as random factor were used. The method of Scheffé was used to correct for multiple testing.

Based on scientifically recognized thresholds (low risk for osteoradionecrosis at D_mean_ below 40 Gy, moderate risk 40–50 Gy, high risk above 50 Gy) [8], [11], a decision tree with treatment recommendations for clinicians was built. For this purpose, the upper limit of the 95% confidence interval of D_mean_ was considered.

The statistical analysis was performed using STATA (Version 17.0, College Station, TX, USA) with a significance level of 5%.

## Results

Data of 112 patients (88 male, 24 female, mean age 63.5 ± 10.3 years) were included (for detailed group assignment see Fig. 1).

**Fig. 2:**
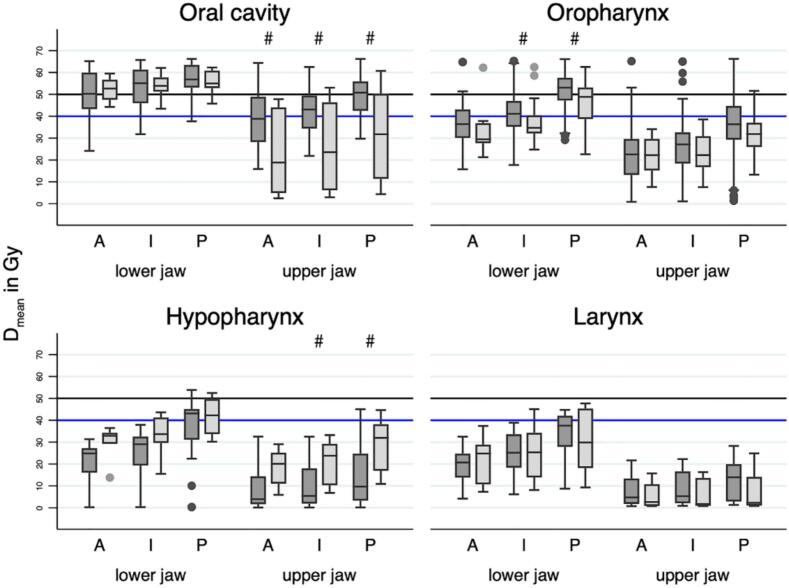
Boxplots representing the calculated D_mean_ according to tumour location, radiotherapy concept, jaw, and dental region. Dark grey: definitive radiotherapy, light grey: adjuvant radiotherapy; dental region A: anterior region (incisors), I: intermediary region (canines, premolars), P: posterior region (molars). Blue line demarcates the threshold of radiation exposure for low risk (< 40 Gy), and the black thick line for high risk (> 50 Gy) for severe side effects. Significant differences between concept of radiotherapy (adjuvant and definitive) within one dental region, tumour location, and jaw are given by a hashtag (#), differences between jaws within one dental region, tumour location, and radiotherapy concept were all significant.

The mean radiation exposure (D_mean_) significantly varies between the dental regions within the jaw depending on the radiotherapy concept and on the primary tumour location (Table 1, Fig. 2).

**Table 1:** Mean values ± Standard deviations (SD) of D_mean_ in Gy for the different tumour locations differentiated by radiation concept (definitive or adjuvant), jaw (upper and lower jaw) and dental region (anterior region [incisors], intermediary region [canines, premolars], posterior region [molars]). N: number of assessed areas subsumed over the patients per tumour location. Significant differences between tumour locations within one dental region, jaw and radiotherapy concept is given by different lower-case letters. Significant differences between dental regions within one jaw, concept of radiotherapy and tumour location is given by different upper-case letters.

Jaw	Dental region	Oral cavity				Oropharynx				Hypopharynx				Larynx			
		Definitive		Adjuvant		Definitive		Adjuvant		Definitive		Adjuvant		Definitive		Adjuvant	
		N	Mean ± SD	N	Mean ± SD	N	Mean ± SD	N	Mean ± SD	N	Mean ± SD	N	Mean ± SD	N	Mean ± SD	N	Mean ± SD
Upper	anterior	14	^a^ 38.7 ± 14.0 ^A^	8	^ab^ 23.2 ± 20.1 ^A^	37	^b^ 22.6 ± 13.7 ^A^	16	^a^ 21.9 ± 8.2 ^A^	16	^c^ 8.9 ± 10.2 ^A^	5	^ab^ 18.2 ± 9.6 ^A^	10	^c^ 8.3 ± 7.9 ^A^	6	^b^ 5.6 ± 6.3 ^A^
	intermediary	28	^a^ 42.1 ± 11.1 ^A^	16	^ab^ 25.6 ± 20.0 ^A^	74	^b^ 25.7 ± 14.4^B^	32	^a^ 23.4 ± 8.2 ^A^	32	^c^ 9.6 ± 9.9 ^A^	10	^ab^ 21.0 ± 10.4 ^A^	20	^c^ 8.8 ± 7.7 ^A^	12	^b^ 6.2 ± 6.6 ^A^
	posterior	28	^a^ 49.2 ± 9.5^B^	16	^a^ 31.3 ± 20.2^B^	74	^b^ 34.9 ± 16.6^C^	32	^a^ 32.4 ± 9.4^B^	32	^c^ 14.6 ± 14.4^B^	10	^a^ 29.0 ± 12.2^B^	20	^c^ 12.7 ± 9.4^B^	12	^b^ 7.8 ± 8.8 ^A^
Lower	anterior	14	^a^ 49.4 ± 12.2 ^A^	8	^a^ 52.2 ± 5.7 ^A^	37	^b^ 36.6 ± 11.0 ^A^	16	^b^ 32.6 ± 9.3 ^A^	16	^c^ 21.1 ± 9.2 ^A^	5	^b^ 29.3 ± 9.1 ^A^	19	^c^ 19.8 ± 8.7 ^A^	6	^b^ 22.3 ± 11.3 ^A^
	intermediary	28	^a^ 52.8 ± 10.1 ^A^	16	^a^ 53.7 ± 5.5 ^A^	74	^b^ 41.3 ± 11.0^B^	32	^b^ 36.5 ± 8.6^B^	32	^c^ 25.5 ± 10.1^B^	10	^b^ 32.7 ± 9.5 ^A^	20	^c^ 24.8 ± 9.9^B^	12	^b^ 25.0 ± 12.3 ^A^
	posterior	28	^a^ 56.3 ± 8.0^B^	16	^a^ 55.7 ± 5.4 ^A^	74	^a^ 51.6 ± 9.0^C^	32	^b^ 46.8 ± 8.7^C^	32	^b^ 36.9 ± 13.6^C^	10	^b^ 42.0 ± 8.4^B^	20	^b^ 33.0 ± 11.5^C^	12	^b^ 30.2 ± 15.0 ^A^

The comparison between adjuvant and definitive radiotherapy and calculated over all dental regions, revealed for carcinomas in the oral cavity significantly lower D_mean_ (p = 0.024) in case of adjuvant therapy; for tumours in the hypopharynx D_mean_ was significantly higher in case of adjuvant therapy (p = 0.034). For all other tumour locations, no significant differences were found between adjuvant and definitive radiotherapy.

For both the adjuvant and the definitive therapy concept, significant differences were found between the tumour locations independent of dental region (p < 0.0001). In the definitive therapy groups, all comparisons showed significant differences except for the tumour locations larynx vs. hypopharynx. In the adjuvant therapy groups, significant differences were only found for larynx vs. oral cavity (p < 0.001), and between larynx and oropharynx (p = 0.002).

Independent of the tumour location, significantly higher D_mean_ was found for the lower jaw compared to the upper jaw (p < 0.001). The comparison between dental regions within one jaw revealed significant differences between most of dental regions in the lower jaw; fewer differences were found in the upper jaw; in particular, no differences were found between the anterior region and the intermediary.

**Fig. 3:**
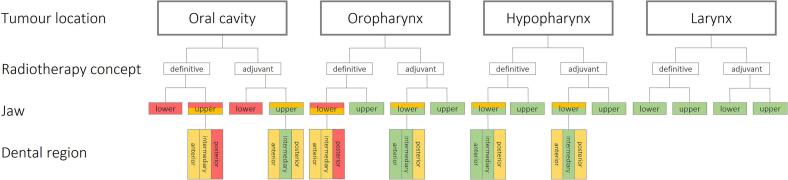
Decision tree to determine the dental treatment requirements prior to radiotherapy. Green: radiation exposure < 40 Gy, low risk, no different treatment planning necessary compared to healthy persons, yellow: radiation exposure 40–50 Gy, moderate risk, individual planning and decision making necessary, red: radiation exposure > 50 Gy, high risk, radical treatment planning including tooth extractions in case of inflammatory processes according to local guidelines. (For interpretation of the references to colour in this figure legend, the reader is referred to the web version of this article.)

Due to the differences found between locations of tumour, concept of radiotherapy and dental region, it was possible to create a decision tree usable to determine the dental treatment requirements prior to radiotherapy (Fig. 3). Green represents that there is a low risk for development of severe sequelae and that treatment decisions can be made that correspond to healthy patients not requiring radiotherapy. Red means that radical treatments including extraction of teeth have to be implemented in case of inflammatory processes according to local guidelines. In case of yellow, individual planning is required with special focus on the apical and periodontal aspects of the teeth.

## Discussion

The first objective of the study was to determine whether radiation exposure differs between different tumour locations, radiotherapy concepts, jaws and dental regions with the second aim to create a decision-making model (decision tree) to facilitate dental rehabilitation prior to radiotherapy. The mean radiation exposure varied considerably between the subgroups and no recommendation can be given applicable to all patients. Significant differences were found between the tumour locations independent of dental region and radiotherapy concept with highest D_mean_ occurring in tumours of the oral cavity and the lowest in tumours in the larynx region. Overall, significantly higher D_mean_ was found in the lower jaw than in the upper jaw and in the posterior than in the anterior region. These findings have a clear impact on dental treatment decisions prior to radiotherapy and the current recommendations regarding extraction strategies should be reconsidered depending on the risks for side effects. The widespread use of concurrent chemotherapy and any comorbidities can further increase the risk of osteoradionecrosis [18]. However, the impact of chemotherapy or comorbidity is not the focus of this study. According to the guidelines on supportive therapy for oncological patients [19], [20] an excellent oral hygiene, dental restoration under special precautions, functional rehabilitation with maximum protection of the mucosa, and special measures during dental, oral and maxillofacial surgery after radiotherapy are necessary to prevent osteoradionecrosis. However, no recommendations are made that take into account the specific radiation doses in the different dental regions depending on the tumour location. The radiation exposure in the jaw varies depending on the location of the tumour, the lower jaw is generally exposed to higher levels of radiation than the upper jaw and the highest incidence rate of osteoradionecrosis is found with 8% in tumours of the oral cavity [21]. The present study is the first examining radiation exposure in different dental regions. From a dental perspective, this differentiation is essential for individual planning of dental rehabilitation in the sense of precision medicine. Without knowledge of the specific region dependent radiation dose, dental rehabilitation will continue to result in radical tooth extraction, often leading to severely impaired masticatory function, aesthetic appearance and quality of life. In addition to the higher risk of osteoradionecrosis associated with extractions [21], conventional or implant-supported prosthetic rehabilitation are required after tooth extraction in most cases, which also carries risks. Due to higher complication rates of dental implants in irradiated patients (osteoradionecrosis, *peri*-implantitis, failure), conventional prosthetic restorations such as removable dentures are preferred [22], [23]. However, removable dentures can cause pressure points, which in turn can increase the risk of osteoradionecrosis [24]. For this reason, avoiding tooth extraction is the best option to prevent osteoradionecrosis, to keep the quality of life, and the ability for a sufficient nutrition.

The thresholds used to distinguish between low, moderate, and high risk for osteoradionecrosis of the jaw are widely used [25], [26]. In a recent *meta*-analysis [25], radiation doses above 50 Gy correlated with osteoradionecrosis development. Regarding the range of the results in the present study (Fig. 2), it is noticeable that the variability of values is low for tumours in the larynx region, whereas tumours of the hypopharynx have the highest variability and the most outliers. Even though the median is below the threshold for high risk for most dental regions, the individual risk for osteoradionecrosis can be significantly higher in a single patient. In dental practice, the radiation therapist can be consulted in unclear cases and the specific radiation doses can be discussed on the basis of the planning CT bearing the high variability in results and the potential higher risk for individuals in mind. There is a fundamental lack of basic research on the individual pathomechanisms in osteoradionecrosis cases.

A high degree of certainty is required, particularly regarding the decision tree from which clinical recommendations are derived. In a retrospective study [27] on patients with oropharyngeal cancer treated with intensity-modulated radiotherapy, 68 cases with osteoradionecrosis were compared with 131 matched controls. It was demonstrated that the mandibular mean dose was significantly higher in the osteoradionecrosis group compared to the control group. However, the maximum doses did not differ significantly between the groups. Therefore, the average radiation dose (D_mean_) was used not only in most studies [25], [28], but also in the present one as it appears to be the more reliable predictive value for radiation exposure.

To increase the clinical certainty of the decision-making model, the upper limit of the 95% confidence interval of the D_mean_ was used in order to keep the uncertainty low with a concomitant usability of the decision tree. The choice of 95% for the confidence interval is very conservative, as higher values for D_mean_ can only be expected with a probability of 2.5%. In some subgroups, the standard deviation is large due to the small number of cases. Since smaller confidence intervals are to be expected in a study with more patients, some tooth regions that were classified now as critical could shift to a less critical radiation dose. Nevertheless, uncertainty remains regarding stochastic tissue damage in addition to individual responses to radiation exposure.

The decision tree reveals a division into two categories according to the anatomic level affected: (I) Lower anatomic level (larynx and hypopharynx): no high-dose regions and only some dental regions showing a moderate risk for osteoradionecrosis making a radical tooth extraction in these regions questionable. (II) Upper anatomic level (oropharynx and oral cavity): higher radiation doses, often above 50 Gy for tumours of the oral cavity and in the posterior dental region of the lower jaw in case of hypopharyngeal tumours; therefore, higher risk for severe side effects resulting in recommendation of radical treatment planning including extractions of teeth with inflammatory processes according to local guidelines.

Our findings may contribute to reduce tooth extraction prior to radiotherapy. According to our decision tree (radiation exposure < 50 Gy in 40 of 48 dental regions), radical tooth extraction might be no longer necessary in most dental regions or should at least be critically questioned. If this less radical extraction strategy is incorporated into a preventive dental care concept, the incidence of osteoradionecrosis might be reduced [29]. In addition, in a recent *meta*-analysis taking the timing of tooth extractions into account, the incidence of osteoradionecrosis events was calculated in 36′294 patients undergoing radiotherapy. The pooled incidence of osteoradionecrosis did not differ between patients having dental extractions prior to (5.5%) or after radiotherapy (5.3%). Therefore, in dental regions with moderate risk radical tooth extractions could be classified as a kind of over-treatment.

One limitation of the study is the high number of excluded patients. Considering eligibility criteria, most exclusions of participants were due to edentulism. The exclusion of edentulous patients was necessary because no differentiation between the dental regions would have been possible. It might be that due to exclusion of these patients some information has not been considered; however, the dimension of bias most likely would have been larger in case of including edentulous patients. Based on the high number of exclusions, the small number of patients in some subgroups (adjuvant approach in patients with tumours of the larynx or hypopharynx) is another limiting factor of the study. Since surgical resection is mutilating in these locations, radiotherapy is usually used as monotherapy and as a definitive therapy concept. Furthermore, only patients with squamous cell carcinoma have been included in order to increase homogeneity of radiotherapy. Future research should verify our findings and the recommendations of the decision tree in a larger sample, taking into account the radiation of other tumour entities. In addition, dosimetric data should be correlated with clinical data like cases of osteoradionecrosis in a prospective study design in order to increase the reliability of the decision tree.

## Conclusion

The location of the tumour is a determining factor in the radiation exposure of the various jaw regions. The findings of the study presented herein suggest that it may be possible to estimate the regions in which radical tooth extraction strategies are unnecessary prior to the commencement of radiotherapy. In individual cases, consultation with the radiotherapist can provide clarity.

## CRediT authorship contribution statement

**Ingmar Staufenbiel:** Conceptualization, Methodology, Supervision, Visualization, Writing – original draft, Writing – review & editing. **Knut Adam:** Conceptualization, Methodology, Supervision, Visualization, Writing – review & editing. **Marco Flohr:** Data curation, Methodology, Supervision, Writing – review & editing. **Joern Wichmann:** Data curation, Methodology, Supervision, Writing – review & editing. **Hans Christiansen:** Conceptualization, Methodology, Supervision, Writing – review & editing. **Nadine Schlueter:** Supervision, Visualization, Writing – original draft, Writing – review & editing. **Liv Julia Clare Moog:** Data curation, Investigation, Methodology, Writing – review & editing. **Robert Blach:** Writing – review & editing. **Kirstin Vach:** Formal analysis, Writing – original draft, Writing – review & editing. **Roland Merten:** Writing – original draft, Writing – review & editing.

## Declaration of Competing Interest

The authors declare that they have no known competing financial interests or personal relationships that could have appeared to influence the work reported in this paper.
