# Supplementary material for: Prediction of high-dose regions in the jaw as a basis for decision-making in dental rehabilitation prior to radiotherapy in the head and neck area
Source: Clin Transl Radiat Oncol. 2026 Mar 21;59:101152. doi: 10.1016/j.ctro.2026.101152 (PMC13084573; doi:10.1016/j.ctro.2026.101152)
Supplement: Supplementary Data 1 [file mmc1.docx]

| Contouring of the dental regions depending on dental region and on presence or absence of remaining teeth | | |
| --- | --- | --- |
| definition of dental regions | The molars were assigned to the posterior region, the premolars and canines to the intermediary and the incisors to the anterior region. The contoured Volumes of Interest (VOI) were 2.3 cm^3^ in size on average. | |
| fully toothed  single tooth gaps | In the transversal view, the entire width of the bone was included in the delineation.  The root tips were chosen as the vertical limit. If these no longer existed, the maxillary sinus was used as a guide in the upper and the submandibular fossa in the lower jaw.  The teeth or the remaining alveoli after extraction were used as orientation for the mesio-distal limit. In the case of single tooth gaps, the gap was regarded as a tooth itself and included in the respective dental region. | |
| one remaining tooth in one dental region | If more teeth were missing but at least one tooth of a dental region was present, this was taken as a reference for the respective other teeth of this group, except in the upper front. | |
| toothless in one dental region | In the case of no teeth in one dental region, the opposite side was mirrored and the contouring of the corresponding region was conducted as mentioned above.  Since the canines in the upper jaw form the eminentia canina, the area between these structures can be taken into account for the contouring of the anterior region in the upper jaw, if this region was toothless. | |
| minimal remaining teeth in one jaw | If only the minimum of teeth were left, the average mesio-distal widths were used for all missing teeth [5,18]. In these cases, the incisive foramen defined the centre of the upper and the mental protuberance of the lower jaw. |  |
| no remaining teeth in one jaw | Exclusion |  |

Supplementary Table 1: Detailed description of the contouring process depending on the remaining teeth

Supplementary figures


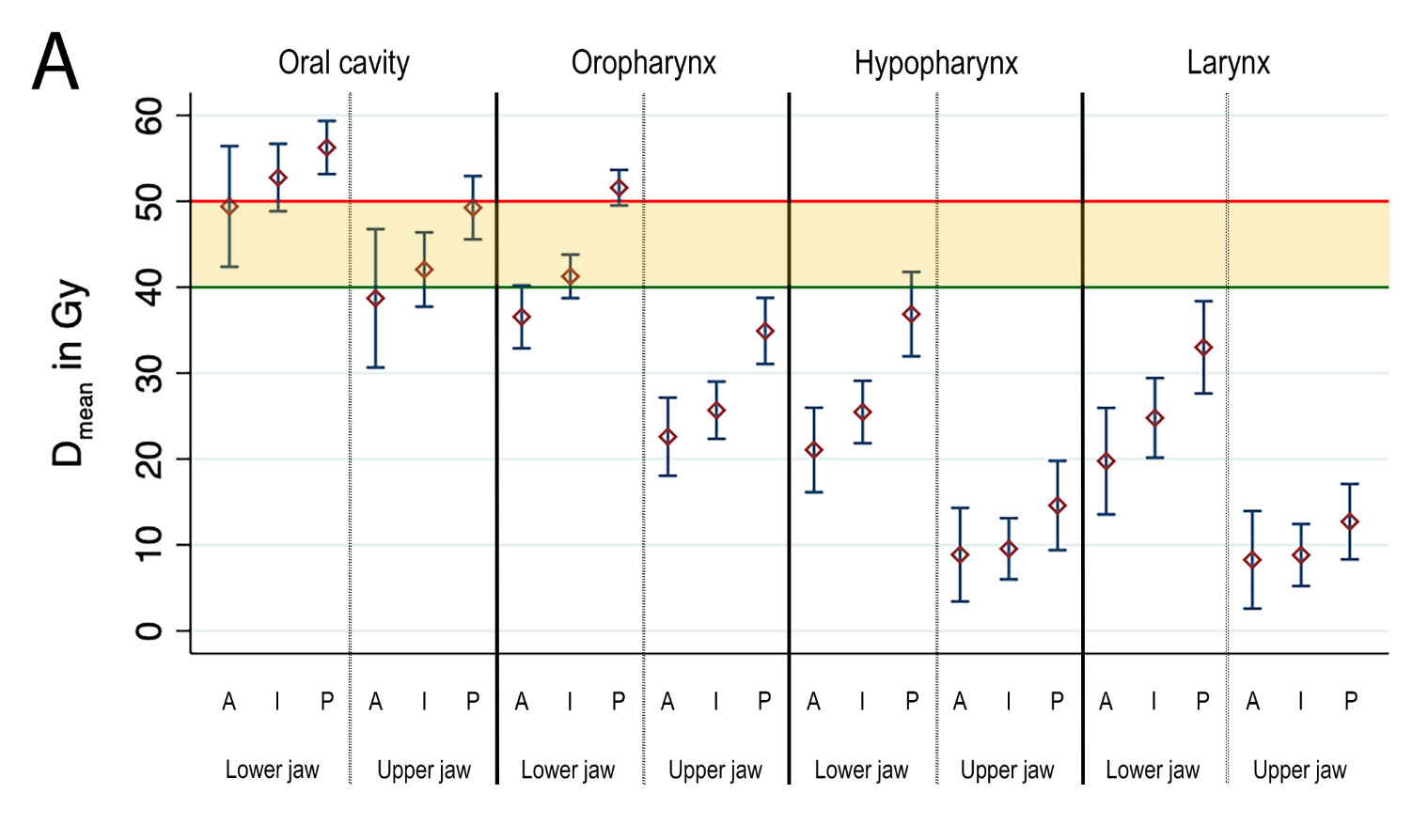

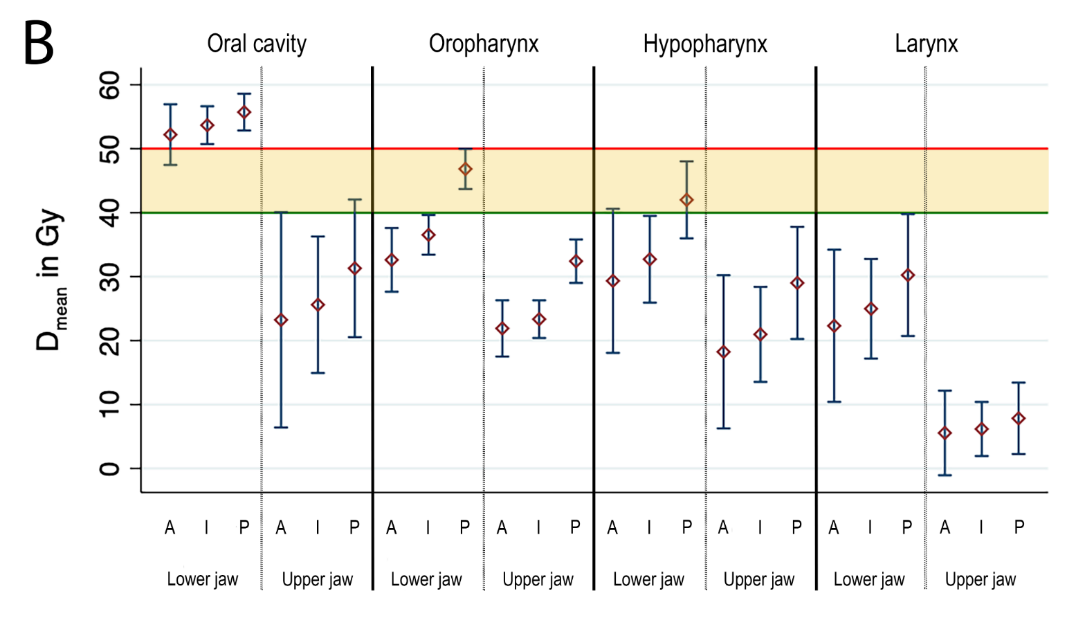


Figure Supp1: Mean values and 95% confidence intervals stratified by tumor location, jaw, and dental region (A: anterior region [incisors], I: intermediary region [canines, premolars], P: posterior region [molars]) for definitive (Figure A) and adjuvant (Figure B) radiotherapy concepts. The decision tree is based on these results. Dental regions in the decision tree are marked green/orange/red if the upper limit of the 95% CI lies below the green line/yellow area between the green and red line/above the red line.
